# Emotional and Uncontrolled Eating Mediate the Well-Being–Adiposity Relationship in Women but Not in Men

**DOI:** 10.3390/nu18010111

**Published:** 2025-12-29

**Authors:** Maria Diez-Hernández, Isabella Parilli-Moser, María Fernanda Zerón-Rugerio, Maria Izquierdo-Pulido

**Affiliations:** 1 Nutrition and Food Safety Research Institute (INSA-UB), Torribera Campus, University of Barcelona, 08921 Santa Coloma de Gramenet, Barcelona, Spain; mariadiez@ub.edu (M.D.-H.); iparillim@ub.edu (I.P.-M.); fernanda.zeron@ub.edu (M.F.Z.-R.); 2 Department of Nutrition, Food Science and Gastronomy, Food Science Torribera Campus, University of Barcelona, 08921 Santa Coloma de Gramenet, Barcelona, Spain; 3 Department of Fundamental and Clinical Nursing, Faculty of Nursing, University of Barcelona, 08907 Hospitalet de Llobregat, Barcelona, Spain

**Keywords:** eating behavior, well-being, diet-quality, obesity, adiposity, sex differences

## Abstract

**Background/Objectives**: Sex and gender influence dietary habits, eating behaviors, mental health, and obesity risk. Women exhibit a higher prevalence of emotional eating and mental health problems, which may contribute to sex-specific differences in adiposity. This study aimed to explore the associations between adiposity, diet quality, eating behaviors, mental health, and well-being, and to examine whether eating behaviors mediate the relationship between mental health and adiposity, stratified by sex. **Methods:** One hundred twenty-three adults (35.6 ± 7.9 years; 63.4% women) with overweight and obesity participated in this cross-sectional study. Adiposity parameters (BMI, body fat, waist and hip circumferences), biochemical parameters, eating behaviors (Three-Factor Eating Questionnaire-R21), well-being (WHO-5), perceived stress (Perceived Stress Scale), diet quality (17-item MedDiet questionnaire), and physical activity (International Physical Activity Questionnaire) were evaluated. Linear regression and path analyses were used to examine associations and mediation effects. **Results:** Women reported higher emotional eating and cognitive restraint scores (*p* = 0.017 and *p* = 0.034, respectively) and greater adherence to the Mediterranean diet (*p* < 0.001) than men. In men, well-being was positively associated with diet quality, while higher stress, cognitive restraint, and poorer diet quality were linked to greater adiposity. In women, well-being and diet quality were inversely associated with adiposity, while emotional and uncontrolled eating were related to higher adiposity and poorer biochemical markers. Emotional and uncontrolled eating mediated the relationship between well-being and adiposity only in women. **Conclusions:** Our results underscore the importance of incorporating sex- and gender-sensitive approaches in obesity prevention and treatment. For women, interventions should focus on emotional regulation and coping strategies, whereas for men, improving diet quality may be more effective.

## 1. Introduction

Sex and gender are important determinants of health and well-being [1]. While sex is usually categorized as female or male and refers to a set of biological attributes, gender encompasses the socially constructed roles, behaviors, expressions, and identities of female, male, and gender-diverse individuals. These social constructs influence how people perceive themselves, behave, and interact within society [1]. It is therefore not surprising that sex and gender differences also influence what and how people eat [2], potentially serving as key determinants of overweight and obesity risk in the general population [3]. For instance, women are more likely to report eating smaller and more frequent meals, while men tend to consume larger portions and skip meals [2]. Furthermore, women tend to experience greater hunger in the morning, while men report higher hunger levels before dinner. Such differences may be explained, at least in part, by sex-based variations in physiological, endocrine and neural factors that can shape eating behaviors [2,4].

Another factor that may contribute to sex and gender differences in eating habits and behaviors is mental health. Importantly, mental health encompasses more than the mere absence of mental disorders. According to the World Health Organization (WHO), it is a state of well-being in which individuals recognize their own abilities, cope with the normal stresses of life, work productively, and contribute meaningfully to their communities [5]. Notably, women are two to three times more likely than men to experience mental health problems, including anxiety and mood-related disorders [4]. Given that food intake can serve as a coping mechanism for negative emotions—a behavior known as emotional eating [6]—it is plausible that women may be more prone to engage in emotional eating than men. Moreover, emotional eating has been associated with obesity, which in turn increases the likelihood of experiencing negative emotions such as anxiety, depression, and stress [7,8], potentially creating a vicious cycle. However, while poor mental health has been consistently linked to emotional and uncontrolled eating behaviors [9,10], the relationship between cognitive restraint (the conscious restriction of food intake to control or reduce weight) and mental health-related variables remains less explored.

The interest of studying the impact of sex and gender differences on dietary intake and eating behaviors, as well as their impact on obesity, arises from the limited effectiveness of current obesity strategies [2,11]. Therefore, exploring additional health determinants, such as sex and gender, can offer valuable insights into this complex public health problem [12,13]. In this context, it is important to recognize that obesity has a multifactorial etiology. As such, it is not solely influenced by dietary habits or levels of physical activity, but also aspects like eating behaviors, biological sex differences, gender-related factors, and mental health can play a fundamental role in its development [13]. Despite this, the relationship between obesity, sex and gender differences, mental health and overall well-being has been less investigated, highlighting the need for further research in these areas [14].

Considering the above, this cross-sectional study aimed to examine the associations between adiposity parameters, dietary intake, eating behaviors, mental health and overall well-being, stratified by sex in adults with overweight/obesity. Additionally, we evaluated whether dietary intake and/or eating behaviors mediate the relationship between adiposity and mental health. We hypothesized that sex differences would influence these associations, with emotional and uncontrolled eating acting as stronger mediators between mental health and adiposity parameters in women than in men. Although both sex and gender are recognized as important determinants of eating behaviors, mental health, and obesity risk, the present study focuses primarily on biological sex differences.

## 2. Materials and Methods

### 2.1. Study Design, Setting, Participants, and Protocol

Adults of both sexes with overweight or obesity were recruited to participate in this cross-sectional study, conducted at the Hospital de la Santa Creu i Sant Pau in Barcelona, Spain. The inclusion criteria were: being men or women with overweight or obesity (BMI from 25 to 35 kg/m^2^), being between 25 and 50 years of age, being metabolically healthy (defined as the absence of diagnosed metabolic diseases and normal clinical laboratory parameters at screening), and being willing to participate in the study. The exclusion criteria were: presence of comorbidities associated with obesity (such as type 2 diabetes mellitus, hypertension, or dyslipidemia), food allergies or intolerances, shift work, diagnosis of an eating disorder, weight loss treatment within the previous 3 months, or a history of bariatric surgery or any bariatric procedure. Additionally, women were excluded if they were postmenopausal, pregnant, or breastfeeding. Recruitment took place in Barcelona and its metropolitan area and included an informational session explaining the study details and inviting volunteers to participate. A total of 245 individuals initially expressed interest in participating. Of these, 112 were excluded during preliminary telephone screening for not meeting eligibility criteria. The remaining 133 were invited to attend an in-person clinical evaluation; however, 9 did not attend, resulting in 124 participants. After applying the full set of inclusion and exclusion criteria and confirming metabolic health status, 123 participants met all eligibility requirements and provided written informed consent (Figure S1). All study procedures were conducted according to the general recommendations of the Declaration of Helsinki and were approved by the Ethics Committee of Drugs Research from the Hospital de la Santa Creu i Sant Pau (Barcelona, Spain) (IIBSP-SOB-2022-109).

### 2.2. Study Variables

#### 2.2.1. Adiposity Parameters

Weight and body composition were measured using a body composition analyzer (InBody 120; Biospace, Seoul, Republic of Korea), with participants wearing light clothing and no shoes. Height was measured to the nearest 0.1 cm using a wall-mounted stadiometer (Seca 217; Seca, Hamburg, Germany). Body mass index (BMI) was calculated as weight (kg) divided by height squared (m^2^).

Waist and hip circumferences were measured to the nearest 0.1 cm using a calibrated flexible steel anthropometric tape (Cescorf, Porto Alegre, Brazil). Waist circumference was measured midway between the lower margin of the rib and the iliac crest, with the subject standing and during gentle expiration [15]. Hip circumference was measured at the level of the greater trochanter, corresponding to the widest portion of the buttocks [15]. The waist-hip ratio was calculated as waist circumference (cm) divided by hip circumference (cm). According to the World Health Organization (WHO), abdominal obesity is defined as a waist-hip ratio (WHR) of at least 0.85 in women and 0.90 in men [15].

#### 2.2.2. Biochemical Analyses

All patients were instructed to attend the study visit after a 12 h overnight fast for venous blood collection. Blood samples were obtained by qualified nursing staff at the Hospital de la Santa Creu i Sant Pau. Samples were subsequently processed and analyzed in the hospital’s chemical laboratory in accordance with standard laboratory procedures. Biochemical parameters related to the glycemic and lipidic profile were analyzed, including glucose, glycated hemoglobin (HbA1c), triglycerides, total cholesterol, high density lipoprotein-(HDL) cholesterol and low-density lipoprotein-(LDL) cholesterol.

Additionally, we calculated the triglycerides-glucose index as a marker of insulin resistance, using the formula: Ln (triglycerides [mg/dL] × glucose [mg/dL]/2) [16].

#### 2.2.3. Diet Quality

Diet quality was evaluated using the 17-item Mediterranean Diet Adherence Screener (MEDAS), which has been validated in the Spanish population [17]. The total score ranges from 0 to 17, with higher scores indicating greater adherence to the Mediterranean Diet.

#### 2.2.4. Eating Behaviors

Eating behaviors were evaluated using the validated Spanish version of the Three Factor Eating Questionnaire (TFEQ-R21) [18], which assesses three dimensions of eating behavior:

- Emotional eating, defined as the tendency to overeat when facing emotionally negative situations, assessed through six items.
- Cognitive restraint, defined as the conscious restriction of food intake to control or reduce weight, assessed through six items.
- Uncontrolled eating, defined as the tendency to overeat in response to a perceived loss of control over food intake, assessed through nine items.

Scores for each dimension were calculated as the mean of the corresponding items, where higher scores indicate greater emotional eating, cognitive restraint, and/or uncontrolled eating [18,19]. In the present population, the TFEQ-R21 demonstrated good internal consistency across all subscales, with Cronbach’s alpha values of 0.901 for emotional eating, 0.810 for cognitive restraint, and 0.863 for uncontrolled eating.

#### 2.2.5. Parameters Related to Mental Health

Well-being was measured using the Spanish version of the WHO-5 questionnaire [20], a self-administered scale to assess the participants’ well-being over the previous two weeks. Total scores range from 0 to 100, with higher scores reflecting greater well-being [21,22]. In our sample, the WHO-5 demonstrated good internal consistency, with a Cronbach’s alpha of 0.812.

Perceived stress was assessed using the Perceived Stress Scale (PSS-10), which has been validated for the Spanish population [23]. This self-reported questionnaire evaluates the degree to which life situations are perceived as unpredictable, uncontrollable, and/or stressful during the previous month. The total score ranges from 0 to 40, with higher scores indicating greater perceived stress [23]. In the present sample, the PSS-10 demonstrated good internal consistency with Cronbach’s alpha values of 0.845.

### 2.3. Covariates

Participant’s sex and date of birth (used to calculate age) were self-reported through standardized questions. Educational level and employment status were assessed using the following questions: ‘What is your education level?’ (Response options: “primary studies” or “more than primary studies”) and ‘What is your current employment status?’ (Response options ‘employed’, ‘student’ or ‘unemployed’).

Physical activity level was assessed in Metabolic Equivalents of Task (METs) using the short version of the International Physical Activity Questionnaire (IPAQ), validated in the Spanish population [24]. Higher MET values indicate greater physical activity intensity.

### 2.4. Statistical Analyses

All analyses were stratified by sex. Normality was confirmed in all variables by Shapiro–Wilks test. Continuous variables are presented as means and standard deviations, whereas categorical variables are expressed as proportions. First, sex differences in study variables were examined using Student’s *t*-test for continuous variables and chi-squared tests for categorical variables. Second, the associations between exposure variables (mental health-related parameters and eating behaviors) and outcome variables (adiposity, biochemical, and lifestyle parameters) were evaluated using linear regression models. Logistic regression analyses were then conducted to examine the odds of adherence to each of the 17-items characterizing the Mediterranean diet as a function of sex (reference group: women), with models adjusted for age and physical activity.

Subsequently, we tested whether the variables significantly associated with mental health-related parameters (i.e., eating behaviors and diet quality) acted as mediators of the relationship between mental health-related parameters and adiposity parameters (Figure S2). Each mediator was analyzed separately using the PROCESS macro (version 3.3) for SPSS [25]. All models were adjusted for age, diet quality, and physical activity, except when the variable was the dependent variable of the model. All statistical analyses were performed using SPSS software version 27 (IBM SPSS Statistics, Chicago, IL, USA). A two-sided *p*-value < 0.05 was considered statistically significant.

## 3. Results

All results are presented stratified by biological sex in accordance with the study objectives. A total of 123 adults (mean age 35.6 ± 7.9 years, 63.4% women) were included in this cross-sectional study. The sociodemographic characteristics of the study population are summarized in Table 1. Overall, most participants held a university degree and were currently employed, with no significant differences observed between sexes.

Regarding adiposity parameters, BMI was similar between men and women (*p* = 0.191). As expected, women had higher body fat percentage (*p* < 0.001) and lower waist circumference than men (*p* < 0.001). Conversely, men showed a higher waist-to-hip ratio than women (<0.001).

In terms of eating behaviors, women showed higher scores for emotional eating and cognitive restraint than men (*p* = 0.017 and *p* = 0.034, respectively), while uncontrolled eating scores were similar between sexes. Similarly, physical activity level did not differ significantly between men and women.

**Figure 1:**
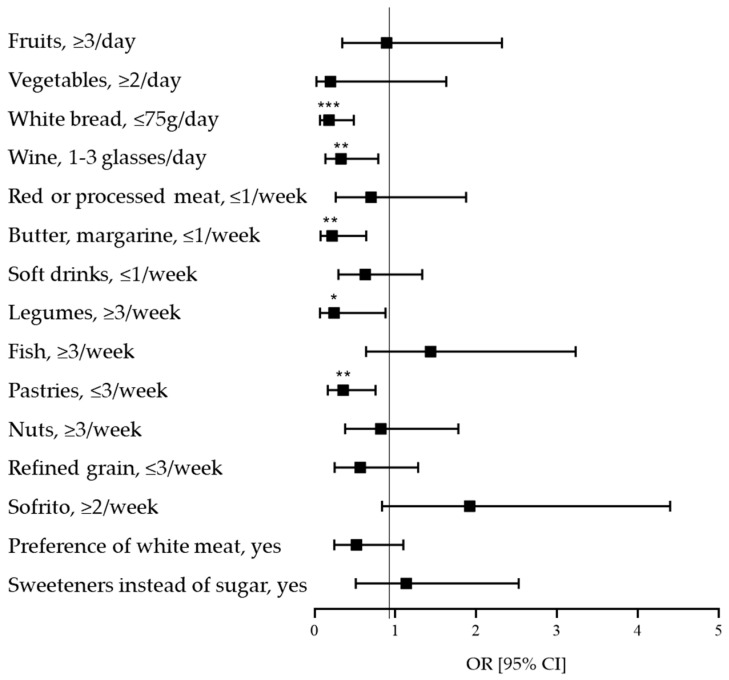
Odds Ratios (ORs) for the items that characterize the adherence to the Mediterranean diet as a function of sex. The figure shows OR [95% confidence intervals (CIs)]. Logistic regression models adjusted for age and physical activity were conducted to test the differences in the adherence to the Mediterranean diet considering ‘women’ as the reference group. * *p* < 0.05, ** *p* < 0.010, *** *p* < 0.001.

As for diet quality, women exhibited higher adherence to the Mediterranean diet than men (*p* < 0.001) (Table 1). Interestingly, logistic regression analyses (Figure 1) revealed that, compared to women, men had significantly lower odds of meeting several Mediterranean diet recommendations. Specifically, men were less likely to meet the recommended daily intake of white bread (OR = 0.18 [95% CI: 0.49; 0.07], *p* < 0.001) and wine (OR: 0.33 [95% CI: 0.79; 0.14], *p* = 0.013), and less likely to meet weekly recommendations for butter/margarine (OR: 0.22 [95% CI: 0.64; 0.07], *p* = 0.006), legumes (OR = 0.24 [95% CI: 0.88; 0.07], *p* = 0.031), and pastries (OR: 0.35 [95% CI: 0.76; 0.17], *p* = 0.007). For butter, margarine, and pastries, lower adherence indicates consumption above the recommended amounts.

Regarding sex differences in parameters related to mental health (Table 1), no significant differences were observed in well-being or perceived stress between men and women. However, a trend toward lower well-being was found among men, although it did not reach statistical significance (*p* = 0.064).

Concerning biochemical parameters (Table 1), men presented higher glucose (*p* = 0.016) and triglyceride levels (*p* < 0.001) as well as a higher triglycerides-glucose index (*p* < 0.001). In contrast, HDL-cholesterol was significantly higher in women compared with men (*p* < 0.001).

### 3.1. Asociations Between Adiposity Parameters, Diet Quality, Eating Behaviors and Stress in Men

In men, mental health-related variables showed significant associations with diet quality and two adiposity parameters (Table 2). Specifically, higher well-being was significantly associated with greater diet quality (*p* = 0.043), whereas higher perceived stress levels were positively associated with BMI (*p* = 0.016) and waist circumference (*p* = 0.037).

Regarding eating behaviors in men (Table 2), all of them were significantly associated with at least one adiposity parameter. Emotional eating was positively associated with waist circumference (*p* = 0.026). Uncontrolled eating showed significant positive associations with BMI (*p* = 0.005), waist and hip circumferences (*p* = 0.003 and *p* = 0.027, respectively), and waist-hip ratio (*p* = 0.018). Meanwhile, cognitive restraint was associated with higher body fat percentage (*p* = 0.048) and hip circumference (*p* = 0.040). Additionally, cognitive restraint was the only eating behavior positively associated with diet quality (*p* = 0.016).

As for diet quality (Table 2), higher adherence to the Mediterranean diet was significantly associated with lower adiposity parameters, including BMI (*p* = 0.031), waist (*p* = 0.028) and hip circumference (*p* = 0.049) and a lower triglycerides-glucose index (*p* = 0.013).

### 3.2. Associations Between Adiposity Parameters, Diet Quality, Eating Behaviors, Well-Being and Stress in Women

In women (Table 3), we observed a greater number of significant associations among well-being, eating behaviors, adiposity, and biochemical parameters compared with men. Well-being was negatively associated with all adiposity parameters [BMI (*p* = 0.019), body fat percentage (*p* = 0.011), waist (*p* = 0.019), and hip circumferences (*p* = 0.017)] and positively associated with HDL-cholesterol (*p* = 0.001). Additionally, higher well-being was significantly associated with lower emotional and uncontrolled eating scores (*p* < 0.001 and *p* = 0.009, respectively). In contrast, perceived stress was associated with higher hip circumference (*p* = 0.019), lower HDL-cholesterol (*p* = 0.023), and higher emotional eating score (*p* = 0.002).

Regarding eating behaviors (Table 3), emotional eating was significantly associated with higher BMI (*p* < 0.001), body fat percentage (*p* < 0.001), waist and hip circumferences (*p* < 0.001 for both), and waist-to-hip ratio (*p* = 0.029). Moreover, emotional eating was positively associated with glucose, triglycerides, and triglycerides-glucose index (*p* = 0.002, *p* = 0.012 and *p* = 0.002, respectively), and negatively associated with HDL-cholesterol (*p* = 0.044). Similarly, uncontrolled eating was significantly associated with higher adiposity parameters [BMI (*p* = 0.001), body fat percentage (*p* = 0.003), waist (*p* = 0.003) and hip circumference (*p* = 0.003)] as well as with biochemical parameters, including triglycerides (*p* = 0.018) and the triglycerides-glucose index (*p* = 0.003). In contrast, cognitive restraint was associated only with lower HbA1c levels (*p* = 0.020).

As for diet quality, higher diet quality score was associated with lower adiposity parameters, including BMI (*p* = 0.003), body fat percentage (*p* = 0.003), waist circumference (*p* = 0.007), and hip circumference (*p* = 0.013).

### 3.3. Emotional and Uncontrolled Eating Mediate the Association Between Well-Being and Adiposity Parameters in Women

**Figure 2:**
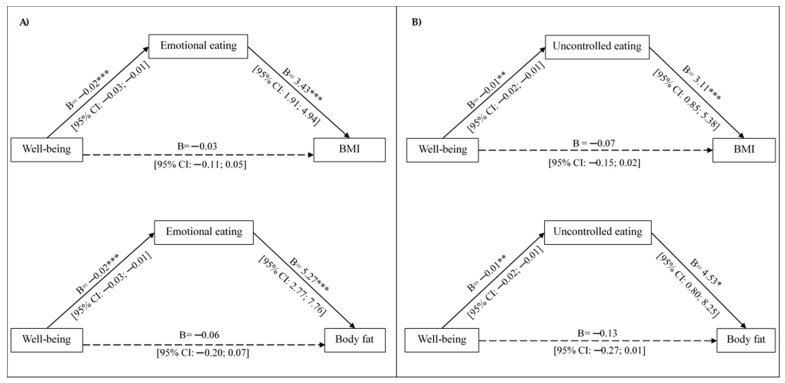
Mediation models illustrating the effect of well-being on adiposity parameters through emotional eating and uncontrolled eating in women. Figure (**A**) shows the indirect effect of well-being on BMI and body fat via emotional eating, and (**B**) via uncontrolled eating. Results are shown as unstandardized B coefficients with their 95% confidence interval. All models were adjusted for age, physical activity, and diet quality. Solid lines indicate statistically significant paths, while dotted lines indicate non-significant paths; * *p* < 0.05, ** *p* < 0.010, *** *p* < 0.001.

Giving that, in women, well-being was associated with eating behaviors, specifically emotional and uncontrolled eating, and that these behaviors were also linked to adiposity parameters, we studied the potential mediating role of these eating behaviors in the relationship between well-being and adiposity parameters (Figure 2A).

The mediation model illustrating the indirect effect of well-being on adiposity parameters via emotional eating is shown in Figure 2A. Both paths a_1_ and b_1_ of the structural model were statistically significant, indicating that well-being was significantly associated with the mediator (emotional eating), and the mediator was significantly associated with BMI and body fat percentage. The direct path (c′) was not statistically significant. Accordingly, our results revealed a significant indirect association between lower well-being and higher BMI and body fat percentage via emotional eating in women (indirect effect = −0.07 [95% CI: −0.15; −0.03] and indirect effect = −0.11 [95% CI: −0.21; −0.05], respectively). Emotional eating explained approximately 38.9% of the variance in BMI and 38.4% in body fat percentage (R = 0.624; R^2^ = 0.389 and R = 0.620; R^2^ = 0.384, respectively; *p* < 0.001). 

Likewise, Figure 2B shows a significant indirect association between higher well-being and lower BMI and body fat percentage via uncontrolled eating (indirect effect = −0.03 [95% CI: −0.07; −0.01] and indirect effect = −0.05 [−0.11; −0.01], respectively). Uncontrolled eating explained approximately 29.1% of the variance in BMI and 29.0% in body fat percentage (R = 0.539; R^2^ = 0.291 and R^2^ = 0.290, respectively; *p* < 0.001).

## 4. Discussion

The main finding of our study is that the associations between well-being, eating behaviors, diet quality, and adiposity are strongly influenced by sex. Among women, greater adiposity was significantly associated with poorer well-being, higher emotional and uncontrolled eating behaviors, and lower diet quality. Furthermore, mediation analyses revealed that, in women only, emotional and uncontrolled eating behaviors mediated the relationship between well-being and adiposity, independently of diet quality and physical activity. Collectively, these results suggest that the mechanisms linking well-being and adiposity differ between men and women, underscoring the need for sex-specific intervention strategies. As an initial approach, we postulate that sex-related factors may partly explain these differences. As such, women tend to be more prone to emotional eating than men, possibly due to differences in the physiological response to stress [26]. Women often exhibit lower hypothalamic–pituitary-adrenal (HPA) axis and autonomic stress responses than men, and since stress activation typically involves HPA axis hyperactivation and appetite suppression [26,27], this could partly explain why women are more likely to use food as a coping mechanism. Additionally, hormonal fluctuations across the menstrual cycle appear to modulate emotional and binge eating, with higher risk during the mid-luteal phase and lower risk during the ovulatory phase, when estradiol levels peak [4]. However, these interpretations should be made with caution, as gender-related psychosocial factors were not directly assessed in the present study.

Furthermore, our findings highlight the role of low well-being in promoting poor eating behaviors—characterized by greater emotional and uncontrolled eating behaviors – which, in turn, are associated with higher adiposity in women but not in men. These results are consistent with the findings of Chlabicz et al. [28], who reported that poorer well-being in women was mainly associated with fat distribution, whereas in men it was more strongly related to muscle mass and, to a lesser extent, fat distribution. Sex and gender-related differences may also be influence by psychosocial factors, as higher BMI is often associated with lower self-esteem, body image dissatisfaction, and reduced self-confidence, particularly in women [29,30,31,32]. Moreover, individuals with greater adiposity frequently experience social pressure to conform to society’s standards of weight and appearance [33], while experiencing prejudice, discrimination, and social stigma in daily life, which may further compromise psychological well-being and overall health [34].

Regarding the role of eating behaviors in the well-being—obesity association, our results revealed that a significant mediation effect of emotional and uncontrolled eating behaviors was present only in women. This finding suggests that women experiencing lower levels of well-being may use food as a coping mechanism and, importantly, may also exhibit greater loss of control over eating (uncontrolled eating behavior), supporting the mediating role of these behaviors in the association between poor well-being and adiposity. It should be noted that the consumption of sweet and fatty foods can temporarily alleviate feelings of distress, which may reinforce these maladaptive eating patterns through reward-related mechanisms [35,36]. Consistent with this interpretation, previous research from our group demonstrated that the consumption of fast-food, commercially baked goods, and sweets is associated with higher emotional and uncontrolled eating scores [37].

Equally interesting is that, beyond adiposity, these maladaptive eating behaviors were significantly associated with adverse metabolic outcomes in women. Specifically, greater emotional eating was significantly associated with higher blood glucose, triglyceride levels and increased insulin resistance. Similarly, greater uncontrolled eating was associated with higher triglyceride levels and insulin resistance, while higher cognitive restraint was related to improved HbA1c. These findings are consistent with previous research reporting that emotional eating was associated with lower HDL-cholesterol and greater insulin resistance only in women, while higher cognitive restraint was linked to a more favorable lipid profile and reduced insulin resistance [38]. Moreover, a prospective cohort study in adolescents with obesity undergoing dietary treatment for weight loss showed that reductions in uncontrolled eating were associated with lower insulin levels, decreases in emotional eating were related to reductions in blood glucose, and increases in cognitive restraint were associated with decreases in insulin and triglyceride levels [39]. Collectively, these results suggest that higher uncontrolled and emotional eating may contribute to greater cardiometabolic risk among women [40].

In men, greater cognitive restraint was significantly associated with higher adiposity, but paradoxically also with better diet quality. Similar associations between cognitive restraint, adiposity, and diet quality have been reported in previous studies conducted by our group in the general population [37,41]. Existing evidence suggests that perceptions of food healthiness may play a role in food choices among individuals with greater cognitive restraint [42,43]. Notably, perceptions regarding the “healthiness” or ‘‘fatteningness’’ of foods can bias estimations of their caloric content, potentially undermining weight management efforts [42]. In fact, experimental studies have shown that young adults who usually choose low-calorie or “healthy” foods for weight control tend to underestimate their caloric content and ultimately consume more calories [42,43]. As a result, greater dietary restraint has often been linked to overweight [37,41,43].

It is worth noting that in our study, associations between cognitive restraint and adiposity in men were observed alongside the relationship between uncontrolled eating and adiposity. This pattern is consistent with the restraint theory, which postulates that chronic dietary restriction alternates with episodes of overeating, thereby promoting weight gain, further restriction and a self-perpetuating cycle of weight fluctuation [18].

Interestingly, despite their lower overall diet quality compared with women (6.5 ± 2.3 points versus 8.0 ± 2.1 points), only men exhibited a significant association between adherence to the Mediterranean diet and higher well-being. This finding aligns with previous evidence showing that the Mediterranean diet supports the synthesis of serotonin, a neurotransmitter closely linked to mood regulation and well-being [44]. Serotonin synthesis depends on the availability of its dietary precursor, tryptophan, which is abundant in Mediterranean diet foods, such as nuts, seeds, whole grains, legumes, fish and dairy products [44]. However, this finding contrasts with previous research reporting a positive association between diet quality and well-being in women [45,46]. In our sample, well-being in women appeared to be more strongly related to adiposity, as previously discussed. This may be explained by current evidence indicating that adiposity can influence the perception of well-being [47,48], an effect that may differ by sex. Together, these findings emphasize the importance of developing sex-specific behavioral strategies to enhance the effectiveness of dietary recommendations.

Our results are consistent with previous evidence indicating that individuals with poorer dietary habits, such as men in our sample, tend to exhibit less favorable cardiometabolic profiles. For instance, Barrea et al. [49] reported that men consumed fewer fruits, fish and shellfish, nuts and sofrito, while consuming more soft drinks and red and processed meats. These dietary differences may partially explain why men showed a poorer glycemic profile (higher glucose levels and insulin resistance) and a less favorable lipid profile (higher triglycerides and lower HDL-cholesterol) compared with women. Consistently, a recent meta-analysis demonstrated that higher adherence to the Mediterranean diet improves both lipid and glycemic profiles and reduces the incidence of cardiovascular diseases [50].

Regarding other mental health-related variables, stress was associated with greater adiposity only in men. This finding is consistent with a study conducted in a Norwegian population that observed a positive association between stress and BMI [7]. Chronic HPA activation and increased cortisol secretion—the main stress hormone—may mediate this relationship by promoting fat accumulation, insulin resistance, and cravings for energy-dense foods [51,52].

Regarding similarities between sexes, our results showed that in both women and men, greater uncontrolled eating was associated with higher adiposity parameters. This may be explained by the fact that uncontrolled eating is related to the consumption of palatable, energy-dense foods rich in sugars and fats, leading to the accumulation of triglycerides in adipose tissue and subsequent metabolic alterations that contribute to overweight and obesity [40]. Although some previous studies reported this association mainly in women [53], our results suggest that uncontrolled eating represents a shared risk factor across sexes.

Taken together, these results emphasize the importance of developing sex-specific strategies to improve the effectiveness of dietary recommendations and nutritional interventions. In women, emphasis should be given to enhancing emotional coping skills and implementing interventions aimed at reducing emotional and uncontrolled eating, given their strong association with adiposity and cardiometabolic outcomes. Conversely, in men, interventions should focus on improving diet quality, while addressing patterns of cognitive restraint and stress that may contribute to weight gain. By targeting these sex-specific behavioral and biological pathways, obesity prevention and weight control programs may achieve greater long-term effectiveness.

This study has certain limitations. First, its cross-sectional design prevents establishing causation. Second, diet quality and physical activity were assessed using self-reported questionnaires, which are subject to underreporting and recall bias. Third the relatively modest sample size, particularly in the male subgroup, may have limited the statistical power to detect small-to-moderate associations. Finally, no additional gender-related variables (such as gender identity, caregiving responsibilities, or socially defined gender roles) were collected, which limits the ability to disentangle biological sex effects from gender-related influences. Despite these limitations, the study also has several strengths, including the use of validated instruments to assess well-being, perceived stress, and eating behaviors. Furthermore, the sex-based analytical approach adds substantial value, as this is, to our knowledge, the first study to report that emotional and uncontrolled eating mediate the association between well-being and obesity-related parameters specifically in women.

## 5. Conclusions

In conclusion, our findings provide novel evidence that the relationships between well-being, eating behaviors, diet quality, and adiposity are strongly influenced by sex. Among women, emotional and uncontrolled eating emerged as key pathways linking poorer well-being with increased adiposity, underscoring the importance of obesity prevention strategies focused on emotional regulation, stress management, and psychological support. In men, by contrast, adiposity was mainly associated with lower adherence to the Mediterranean diet and dietary patterns influenced by cognitive restraint and stress, highlighting the need for nutritional counseling, promotion of healthy dietary patterns, and strategies aimed at improving diet quality and coping with stress. Collectively, these findings highlight the need to develop sex-specific strategies for obesity prevention and treatment in adults, integrating targeted behavioral, emotional, and nutritional components to better inform and enhance clinical practice.

## Figures and Tables

**Table 1:** General characteristics of the population studied stratified by sex (n = 123).

	**Women**	**Men**	***p*-Value**
**Sample size, % (n)**	63.4 (78)	36.6 (45)	
**Sociodemographic variables**			
Age, years	35.4 ± 7.9	36.0 ± 7.8	0.695
Educational level, % university degree	93.6	84.4	0.101
Employment status, % employed	94.9	97.8	0.436
**Adiposity parameters**			
BMI, kg/m^2^	27.7 ± 5.9	29.1 ± 5.6	0.191
Body fat, %	36.3 ± 9.6	27.9 ± 8.7	**<0.001**
Waist circumference, cm	81.1 ± 12.2	96.7 ± 12.2	**<0.001**
Hip circumference, cm	108.5 ± 11.5	109.8 ± 10.0	0.511
Waist-hip ratio, a.u.	0.8 ± 0.1	0.9 ± 0.1	**<0.001**
**Eating behaviors**			
Emotional eating, score	2.1 ± 0.8	1.7 ± 0.6	**0.017**
Uncontrolled eating, score	2.0 ± 0.6	2.1 ± 0.6	0.421
Cognitive restraint, score	2.3 ± 0.6	2.0 ± 0.5	**0.034**
**Lifestyle variables**			
Physical activity, METs	2222.6 ± 1671.9	2111.3 ± 1530.1	0.715
Diet quality, score	8.0 ± 2.1	6.5 ± 2.3	**<0.001**
**Parameters related to mental health**			
Well-being, score	61.6 ± 15.6	56.0 ± 16.5	0.064
Stress, score	14.6 ± 7.0	14.6 ± 4.8	0.999
**Biochemical parameters**			
Glucose, mg/100 mL	79.8 ± 7.4	86.5 ± 22.1	**0.016**
HbA1c, %	5.2 ± 0.3	5.3 ± 0.6	0.144
Triglycerides, mg/100 mL	68.5 ± 38.2	109.3 ± 52.3	**<0.001**
Triglycerides-glucose index, a.u.	7.8 ± 0.5	8.3 ± 0.6	**<0.001**
Total cholesterol, mg/100 mL	184.6 ± 30.4	191.2 ± 33.6	0.270
HDL-cholesterol, mg/100 mL	59.7 ± 12.0	47.5 ± 9.0	**<0.001**
LDL-cholesterol, mg/100 mL	111.0 ± 26.6	120.4 ± 26.8	0.068

a.u., arbitrary unit; BMI, Body mass index; HbA1c, Glycated hemoglobin; HDL, high density lipoprotein; LDL, low density lipoprotein; METs, Metabolic Equivalents of Task. Data are expressed as mean and standard deviation for continuous variables, and as percentages for categorical variables. Differences between outcome variables as a function of sex were tested using Student’s *t*-test for continuous variables and χ^2^ test for categorical variables. Statistically significant *p*-values (*p* < 0.050) are shown in bold.

**Table 2:** Multivariable linear regression models of associations between parameters related to mental health, eating behaviors, diet quality, and adiposity/biochemical parameters in men (n = 45).

	Parameters Related to Mental Health		Eating Behaviors			Diet Quality, Score B [95%CI]
	Well-Being, Score B [95%CI]	Stress, Score B [95%CI]	Emotional Eating, Score B [95%CI]	Uncontrolled Eating, Score B [95%CI]	Cognitive Restraint, Score B [95%CI]	
**Adiposity parameters**						
BMI, kg/m^2^	−0.03 [−0.14; 0.08]	0.43 [0.09; 0.77] *	2.32 [−0.34; 4.98]	3.92 [1.22; 6.61] **	2.92 [−0.44; 6.27]	−0.84 [−1.60; −0.08] *
Body fat, %	−0.06 [−0.23; 0.11]	0.46 [−0.11; 1.03]	1.77 [−2.58; 6.11]	3.53 [−1.05; 8.12]	5.31 [0.05; 10.58] *	−0.86 [−2.06; 0.35]
Waist circumference, cm	−0.02 [−0.25; 0.22]	0.80 [0.05; 1.55] *	6.33 [0.79; 11.87] *	8.90 [3.21; 14.58] **	5.89 [−1.32; 13.09]	−1.82 [−3.44; −0.20] *
Hip circumference, cm	−0.04 [−0.24; 0.17]	0.62 [−0.03; 1.27]	4.71 [−0.16; 9.58]	5.87 [0.71; 11.03] *	6.40 [0.31; 12.50] *	−1.40 [−2.80; −0.01] *
Waist-hip, ratio, a.u.	0.01 [−0.01; 0.01]	0.01 [−0.01; 0.01]	0.02 [−0.01; 0.05]	0.04 [0.01; 0.06] *	0.01 [−0.03; 0.04]	−0.01 [−0.01; 0.01]
**Diet quality**, score	0.04 [0.01; 0.08] *	−0.05 [−0.20; 0.10]	−0.79 [−1.87; 0.29]	−0.48 [−1.69; 0.72]	1.54 [0.30; 2.78] *	-
**Biochemical parameters**						
Glucose, mg/100 mL	−0.02 [−0.46; 0.43]	−0.36 [−1.88; 1.16]	−0.77 [−12.12; 10.58]	−8.93 [−20.82; 2.96]	−5.99 [−20.19; 8.21]	−2.48 [−5.60; 0.63]
HbA1c, %	−0.01 [−0.02; 0.01]	0.01 [−0.04; 0.05]	0.01 [−0.30; 0.33]	−0.24 [−0.57; 0.09]	−0.06 [−0.46; 0.33]	−0.05 [−0.13; 0.04]
Triglycerides, mg/100 mL	0.15 [−0.98; 1.27]	−0.66 [−4.32; 3.00]	15.37 [−11.26; 42.00]	23.88 [−4.44; 52.21]	4.72 [−29.53; 38.96]	−5.90 [−13.34; 1.56]
Triglycerides-glucose index, a.u.	0.01 [−0.01; 0.01]	−0.01 [−0.04; 0.03]	0.08 [−0.20; 0.36]	0.12 [−0.18; 0.42]	0.06 [−0.30; 0.41]	−0.10 [−0.18; −0.02] *
Total cholesterol, mg/100 mL	0.14 [−0.51; 0.78]	−0.74 [−2.92; 1.44]	3.80 [−12.53; 20.13]	−1.40 [−19.04; 16.23]	11.23 [−9.12; 31.59]	−2.98 [−7.47; 1.51]
HDL-cholesterol, mg/100 mL	−0.06 [−0.24; 0.12]	0.22 [−0.37; 0.81]	−1.45 [−5.97; 3.07]	−3.26 [−7.91; 1.40]	−0.16 [−5.90; 5.59]	0.66 [−0.57; 1.90]
LDL-cholesterol, mg/100 mL	0.27 [−0.30; 0.84]	−0.74 [−2.55; 1.06]	5.36 [−8.26; 18.98]	−3.75 [−18.22; 10.72]	15.75 [−0.86; 32.36]	−1.82 [−5.57; 1.93]
**Parameters related to mental health**						
Well-being, score	-	-	−0.39 [−8.47; 7.69]	−0.97 [−9.67; 7.73]	1.82 [−8.37; 12.00]	-
Stress, score	-	-	1.70 [−0.62; 4.02]	1.37 [−1.17; 3.90]	1.69 [−1.28; 4.65]	-

a.u., arbitrary unit; BMI, Body mass index; CI, confidence interval; HbA1c, Glycated hemoglobin; HDL, high density lipoprotein; LDL, low density lipoprotein Associations between exposure variables with outcome variables were tested using multivariate linear regression models. All analyses were adjusted for age, diet quality and physical activity (except when the covariate was the dependent variable of the model). The table shows the unstandardized coefficient (B), and *p*-value associated with each predictor variable. * *p* < 0.050, ** *p* < 0.010.

**Table 3:** Multivariable linear regression models of associations between parameters related to mental health, eating behaviors, diet quality, and adiposity/biochemical parameters in women (n = 78).

	Parameters Related to Mental Health		Eating Behaviors			Diet Quality, Score B [95%CI]
	Well-Being, Score B [95%CI]	Stress, Score B [95%CI]	Emotional Eating, Score B [95%CI]	Uncontrolled Eating, Score B [95%CI]	Cognitive Restraint, Score B [95%CI]	
**Adiposity parameters**						
BMI, kg/m^2^	−0.10 [−0.19; −0.02] *	0.15 [−0.03; 0.33]	3.63 [2.25; 5.01] ***	3.65 [1.47; 5.84] ***	0.66 [−1.48; 2.79]	−0.95 [−1.57; −0.33] **
Body fat, %	−0.18 [−0.32; −0.04] *	0.24 [−0.06; 0.53]	5.75 [3.46; 8.03] ***	5.56 [1.95; 9.17] **	1.85 [−1.63; 5.33]	−1.56 [−2.57; −0.55] **
Waist circumference, cm	−0.21 [−0.38; −0.04] *	0.24 [−0.14; 0.61]	6.89 [3.96; 9.82] ***	6.93 [2.39; 11.48] **	1.13 [−3.33; 5.59]	−1.80 [−3.11; −0.50] **
Hip circumference, cm	−0.21 [−0.37; −0.04] *	0.43 [0.07; 0.78] *	6.59 [3.75; 9.43] ***	6.70 [2.31; 11.10] **	0.63 [−3.69; 4.94]	−1.61 [−2.88; −0.35] *
Waist-hip, ratio, a.u.	−0.01 [−0.01; 0.01]	−0.01 [−0.01; 0.01]	0.02 [0.01; 0.03] *	0.02 [−0.01; 0.04]	0.01 [−0.02; 0.03]	−0.01 [−0.01; 0.01]
**Diet quality**, score	0.01 [−0.02; 0.04]	−0.01 [−0.08; 0.06]	−0.43 [−1.02; 0.16]	−0.58 [−1.43; 0.28]	−0.55 [−1.33; 0.23]	−
**Biochemical parameters**						
Glucose, mg/100 mL	−0.11 [−0.23; 0.01]	0.16 [−0.09; 0.41]	3.33 [1.27; 5.39] **	3.03 [−0.08; 6.14]	−1.75 [−4.63; 1.14]	0.48 [−0.36; 1.32]
HbA1c, %	0.01 [−0.01; 0.01]	0.01 [−0.01; 0.01]	0.07 [−0.01; 0.14]	0.03 [−0.07; 0.14]	−0.11 [−0.20; −0.02] *	−0.02 [ −0.05; 0.01]
Triglycerides, mg/100 mL	−0.07 [−0.68; 0.54]	0.41 [−0.88; 1.69]	13.97 [3.12; 24.82] *	19.14 [3.34; 34.93] *	4.36 [−10.60; 19.32]	−0.75 [−5.06; 3.57]
Triglycerides-glucose index, a.u.	−0.01 [−0.01; 0.01]	0.01 [−0.01; 0.02]	0.22 [0.08; 0.35] **	0.30 [0.11; 0.50] **	0.01 [−0.18; −0.21]	−0.01 [−0.06; 0.05]
Total cholesterol, mg/100 mL	0.30 [−0.15; 0.75]	−0.26 [−1.22; 0.71]	5.42 [−2.99; 13.83]	11.49 [−0.54; 23.52]	−5.78 [−16.96; 5.40]	0.23 [−3.02; 3.47]
HDL-cholesterol, mg/100 mL	0.30 [0.12; 0.48] ***	−0.47 [−0.87; −0.07] *	−3.67 [−7.24; −0.10] *	−2.66 [−7.95; 2.62]	−0.38 [−5.74; 4.97]	0.88 [−0.54; 2.30]
LDL-cholesterol, mg/100 mL	0.01 [−0.38; 0.40]	0.10 [−0.75; 0.94]	5.44 [−1.91; 12.80]	10.03 [−0.51; 20.57]	−7.80 [−18.52; 2.91]	−0.50 [−3.38; 2.38]
**Parameters related to mental health**						
Well-being, score	-	-	−7.45 [−11.41; −3.49] ***	−8.02 [−14.00; −2.04] **	−2.61 [−8.30; 3.08]	-
Stress, score	-	-	3.07 [1.16; 4.99] **	1.14 [−1.81; 4.10]	0.69 [−2.01; 3.39]	-

a.u., arbitrary unit; BMI, Body mass index; CI, confidence interval; HbA1c, Glycated hemoglobin; HDL, high density lipoprotein; LDL, low density lipoprotein Associations between exposure variables with outcome variables were tested using multivariate linear regression models. All analyses were adjusted for age, diet quality and physical activity (except when the covariate was the dependent variable of the model). The table shows the unstandardized coefficient (B), and *p*-value associated with each predictor variable. * *p* < 0.050, ** *p* < 0.010, *** *p* < 0.001.

## Data Availability

The data presented in this study are available on request from the corresponding author. The data are not publicly available due to privacy restrictions.

## Supplementary Materials

The following supporting information can be downloaded at: https://www.mdpi.com/article/10.3390/nu18010111/s1. Figure S1. Flow diagram of participants included in the study according to STROBE guidelines. Figure S2. Path diagram for the total effect of parameters related to mental health on the adiposity parameters and the indirect effect of parameters related to mental health on adiposity parameters through the potential mediation of eating behaviors.
