# Supplementary material for: Emotional and Uncontrolled Eating Mediate the Well-Being–Adiposity Relationship in Women but Not in Men
Source: Nutrients. 2025 Dec 29;18(1):111. doi: 10.3390/nu18010111 (PMC12787970; doi:10.3390/nu18010111)
Supplement: Supplementary file 1 [file nutrients-18-00111-s001.zip › nutrients-4037207-supplementary.pdf]

nutrients

MDPI

**Supplementary Material:**

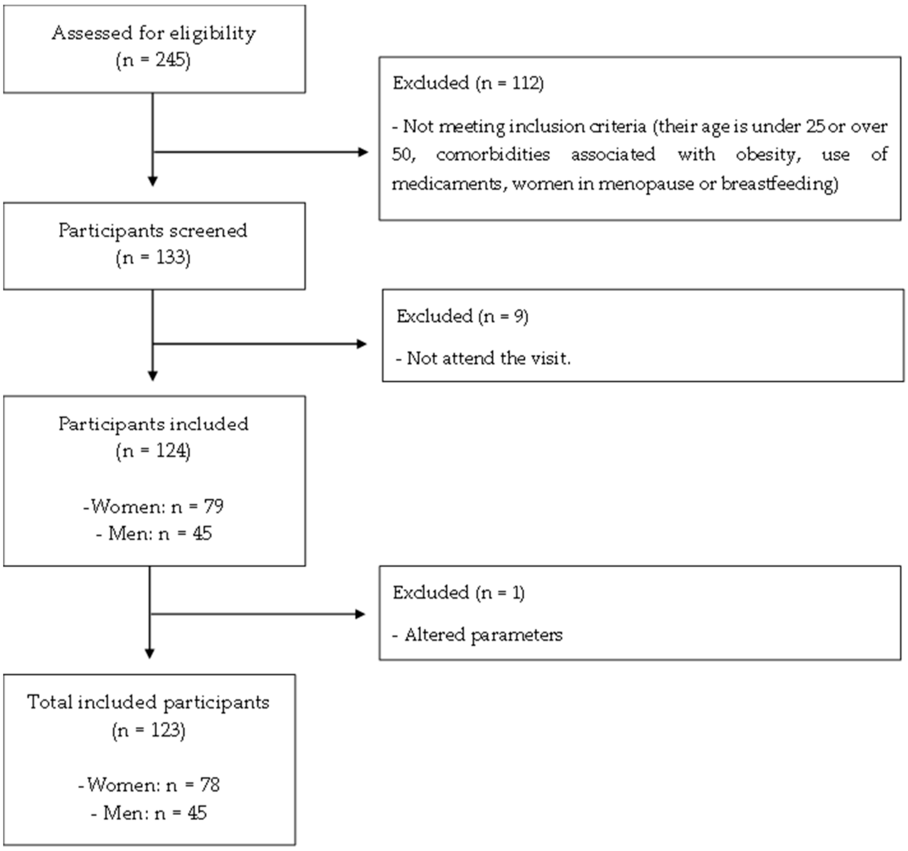


**Figure S1.** Flow diagram of participants included in the study according to STROBE guidelines.

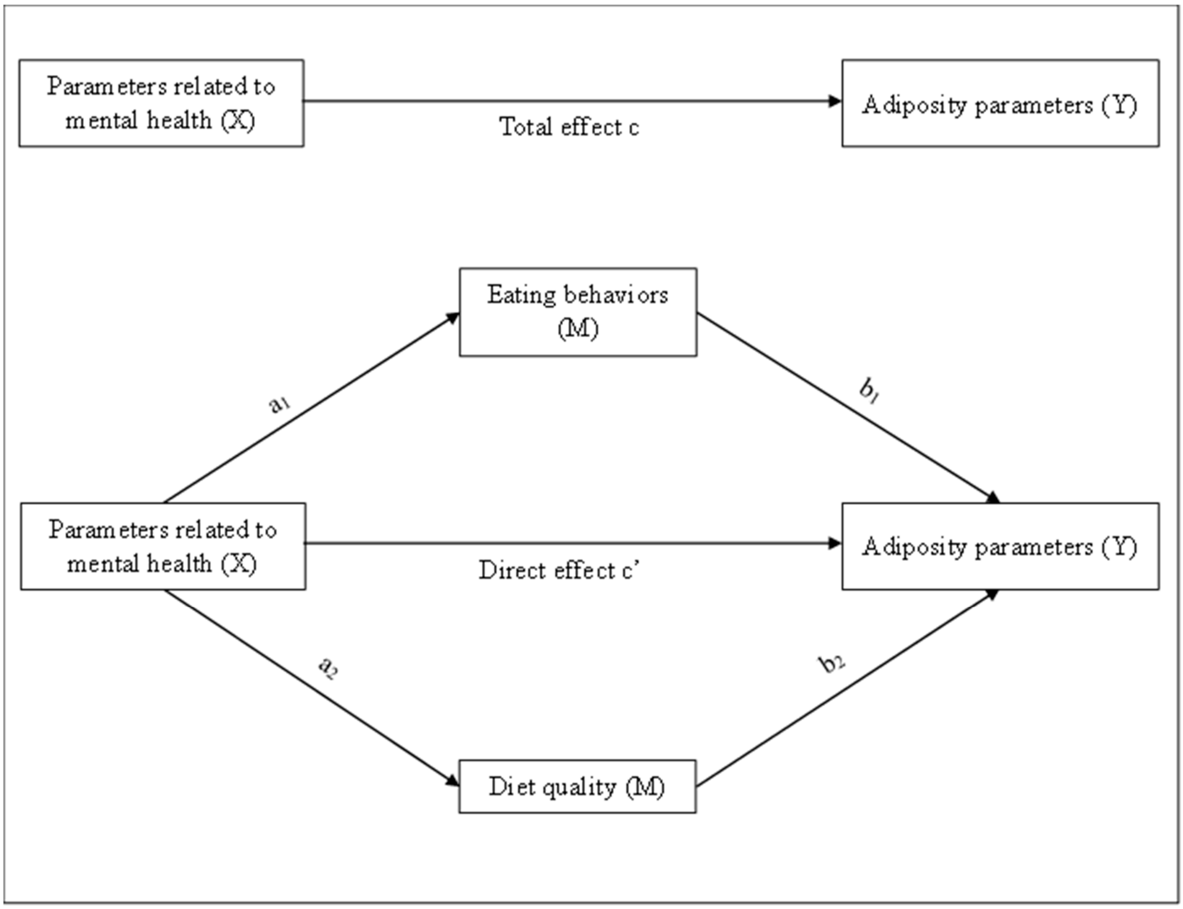


**Figure S2.** Path diagram for the total effect of parameters related to mental health on the adiposity parameters and the indirect effect of parameters related to mental health on adiposity parameters through the potential mediation of eating behaviors. In the top diagram "c" is the total effect of exposure (X) on outcome (Y) ignoring the mediator (M). In the bottom diagrams, the mediation effect of parameters related to mental health on the adiposity parameters through eating behaviors and diet quality is shown, where "a" is the effect of exposure on mediator and "b" is the effect of mediator on outcome. Effect c′ is the direct effect of exposure on outcome while adjusting for the mediator.
